# Crossbar nanoarchitectonics of the crosslinked self-assembled monolayer

**DOI:** 10.1186/1556-276X-9-287

**Published:** 2014-06-09

**Authors:** Hicham Hamoudi

**Affiliations:** 1 International Center for Young Scientists (ICYS), 1-1 Namiki, Tsukuba 305-0044, Japan; 2 International Center for Materials Nanoarchitectonics (WPI-MANA), National Institute for Materials Science (NIMS), 1-1 Namiki, Tsukuba 305-0044, Japan

**Keywords:** Self-assembled monolayer, Crosslinking, Crossbars

## Abstract

A bottom-up approach was devised to build a crossbar device using the crosslinked SAM of the 5,5′-bis (mercaptomethyl)-2,2′-bipyridine-Ni^2+^ (BPD- Ni^2+^) on a gold surface. To avoid metal diffusion through the organic film, the author used (i) nanoscale bottom electrodes to reduce the probability of defects on the bottom electrodes and (ii) molecular crosslinked technology to avoid metal diffusion through the SAMs. The properties of the crosslinked self-assembled monolayer were determined by XPS. *I-V* characteristics of the device show thermally activated hopping transport. The implementation of this type of architecture will open up new vistas for a new class of devices for transport, storage, and computing.

## Background

Self-ordering principle was a basic idea of ancient philosophers: *Only the mutuality of the parts creates the whole and its ability to function*. In the language of chemistry, this means that *the self-organization of molecules engenders supramolecular systems and is responsible for their functions*. The application of self-assembly technology has been extended to surface science during the last two decades. Self-assembled monolayers (SAMs) are highly ordered organic molecular aggregates that are chemisorbed on surfaces with the thickness of a single molecule [1-6]. The conjugate organic SAMs can provide all the ingredients to create new hybrid materials with novel functionalities out of the scope of traditional solid-state devices. This class of molecules exhibits very interesting electronic and magnetic properties such as electron transport by charge injections through different molecular orbitals (MO) [7].

Modification of the conjugate SAMs by electron beam allows fabrication of the crosslinked aromatic SAM [8,9]. Low-energy electrons are necessary to create a crosslinked molecular network. The basic means to form molecular crosslinking is cleavage of the CH bond by the impact of the electrons on the molecular backbone. This phenomenon, for low-energy electrons, dissociative electron attachment (DEA), is generated by the attachment of electrons on the Rydberg states of the molecules, depending on the characteristics of the excitation states in which the electrons are located. This excitation can result in one of two dynamics: (i) simple electron relaxation or (ii) bond rupture that engenders crosslinking phenomena. Modern high-energy electron beam lithography allows the crosslinking of the aromatic molecules and the fabrication of sheets of nanometer size, which also provides evidence that the aromatic self-assembled monolayer acts as a negative electron resist with a high-energy electron beam [8,9].

Metallization of SAMs to design top electrodes is a subject of long-standing interest. Many applications can be found in everyday life. This subject has attracted great attention recently because of interest in organic electronics and light emitting diodes [10]. Metal diffusion into the SAM can drastically alter the properties of the SAM, finally ruining the device because of the formation of filaments or during the evaporation process by which SAMs are chemically altered. Two factors can play an important role in avoiding metal diffusion through SAMs: (i) the quality of the SAM and (ii) the quality of the metal substrate on which a homogeneous surface is put.

The current flowing through junctions composed of assemblies of molecules depends on the energy gap separating the Fermi levels of the electrodes and the valence band of the molecules. A redox-active center (Ni) has been incorporated into the organic backbones to improve the charge-transfer processes. Different studies of molecular redox center immobilized on metallic substrate indicate them as good conductors [11].

As described in this paper, a crossbar device with molecules as interconnecting elements was fabricated on gold bars (100 nm) and thus the use of crosslinked molecular self-assembled monolayer is demonstrated as compatible with use in crossbar nanoelectronic devices. A crosslinked SAM of 5,5′-bis (mercaptomethyl)-2,2′-bipyridine-Ni^2+^ (BPD-Ni^2+^) has been prepared on top of the pre-patterned Au bottom contacts. Then the top Au contacts were evaporated. A two-electrode probe station was used to assess the fidelity of the molecular junctions. Additionally, to elucidate the molecular transport in the device junctions, temperature-dependent *I*-*V* examinations were performed.

## Methods

### Fabrication of the crossbar molecular devices

#### Fabrication of the bottom electrode

**Figure 1:**
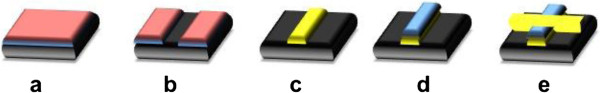
**Scheme process flow for fabrication of crossbar molecular devices. (a)** Photoresist patterning for bottom contacts on SiO_2_. **(b)** The 100-nm bar patterns were created using electron beam lithography. **(c)** Deposition of 10 nm of Ti and 150-nm Au over patterned substrate and lift-off excess Au with photoresist removal. **(d)** Deposition of SAM over the entire substrate. **(e)** Preparation and deposition of top electrodes.

Lithography of bottom electrodes was accomplished by starting with a clean single-side polished SiO_2_ substrate. Photoresist PMMA 950 was spin-coated on SiO_2_ at 2,000 rpm for 90 s and baked at 180°C for 3 min (Figure 1a). Then, to avoid the charge-up of PMMA, 15 nm of conductive polymer (ESPACER 300Z; Showa Denko K.K., Minato, Tokyo, Japan) was spin-coating on the top of the PMMA at 2,000 rpm for 60 s. The 100-nm bar patterns were fabricated using an electron beam lithography system (50 kV, 100 mC/cm^2^; Elionix Co. Ltd., Hachioji, Tokyo, Japan). The resist was developed in MIBK methyl isobutyl ketone + IPA isopropanol 1:3 solution (MIBK-IPA) for 30 to 40 s to remove the irradiated zones and to form a pattern for the bottom electrode bars (Figure 1b). Finally, using electron-beam deposition, 10 nm of titanium and 150 nm of gold were deposited on the photoresist-patterned wafer. The wafer was immersed in acetone to remove the photoresist and the excess metal which adhered on the resist (Figure 1c).

#### Preparation of the crosslinked BPD-Ni^2+^ SAM

**Figure 2:**
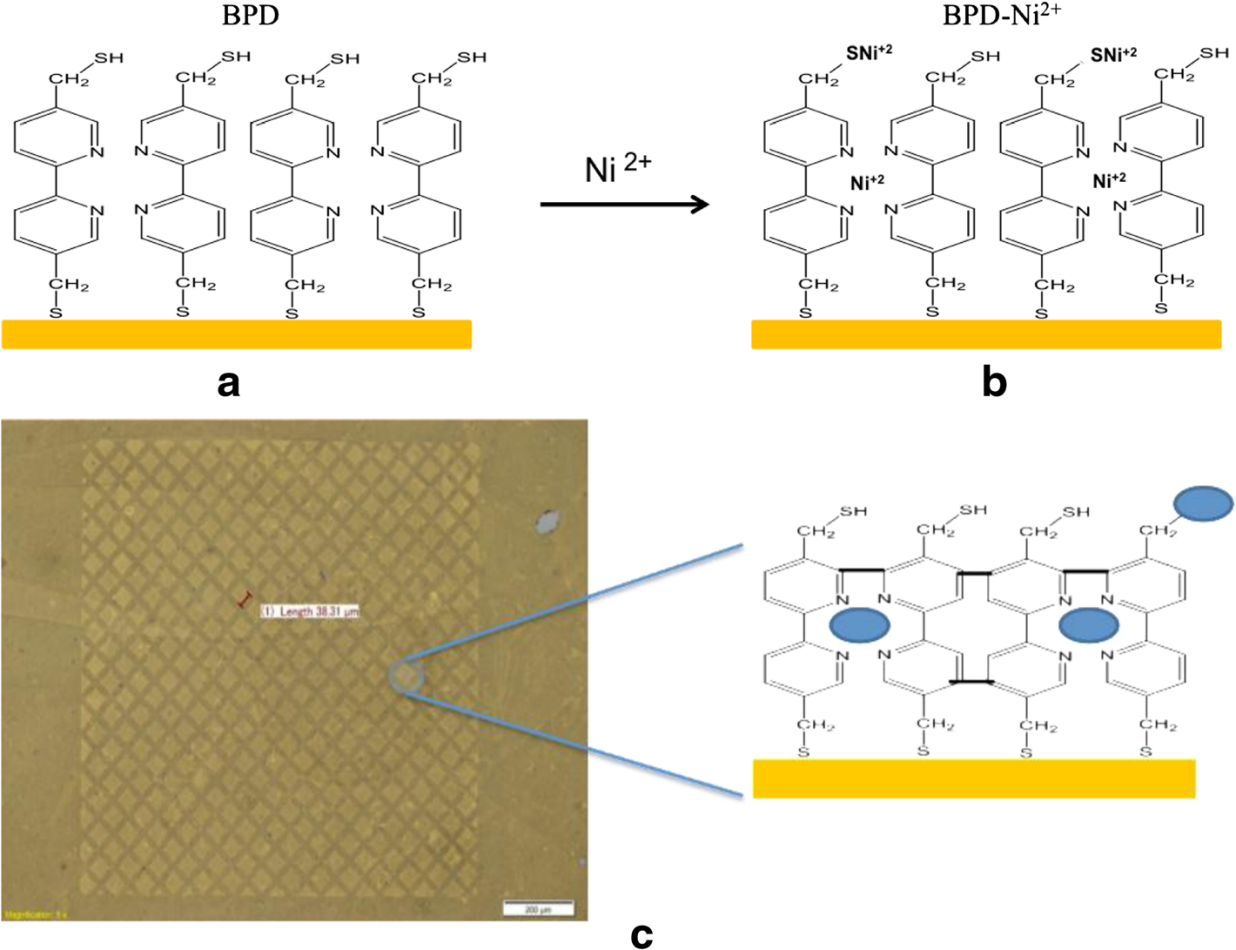
**Preparation of the cross-linked BPD-Ni**^**2+ **^**SAM. (a)** Preparation of the BPD SAM. **(b)** Encapsulation of Ni on the BPD SAM. **(c)** A BPD-Ni system was employed as a negative resist for e-beam lithography. Microscope image of etched BPD-Ni/Au template, preliminary patterned by electrons in proximity printing geometry using a metal mesh as mask. Areas exposed and non-exposed to electrons are marked.

The SAM of BPD films was fabricated in the following manner: 5,5′-bis(mercaptomethyl)-2,2′-bipyridine was purchased from Aldrich and used as received. The SAM of 5,5′-bismercaptomethyl-2,2′-bipyridine (BPD) was prepared by immersing the bottom electrodes in freshly prepared 1-mM solution of *n*-hexane for 1 h at 60°C. Solutions were well-degassed using Ar. All preparation steps were performed in the absence of ambient light, which is the same as the process in our previous studies [4,6]. Subsequently, the bottom gold bar was modified with a layer of BPD and immersed for 3 h in a 50-mM aqueous solution of NiCl_2_ (see Figure 2a,b).

The BPD SAM fabricated as above was characterized using X-ray photoelectron spectroscopy (XPS). XPS spectroscopy measurements were conducted at the MANA Foundry using an XPS spectrometer (Alpha 110-mm analyzer XPS version; Thermo Fisher Scientific, Chiyoda-ku, Tokyo, Japan). The XPS spectra were recorded in the Au 4*f*, S 2*p*, C 1 *s*, N 1 *s*, and Ni 2*p* regions. Spectrum acquisition was done in normal emission geometry using the Al K radiation. The binding energy (BE) scale of each spectrum was calibrated individually to the Au 4*f*_7/2_ emission of an *n*-alkanethiol-covered gold substrate at 83.95 eV. In addition, XPS data were used to ascertain the effective thickness of the target SAMs. This assessment was done based on the Au 4*f* intensity, assuming standard exponential attenuation of the photoelectron signal and using the attenuation lengths described in an earlier report [12].

The exposure of BPD-Ni film to electron beams engenders the formation of crosslinked SAMs. As shown in Figure 2c, the BPD-Ni template was patterned by electrons (50 kV, 60 mC/cm^2^) in proximity printing geometry using a metal TEM mesh as a mask. The patterned template was etched in an I_2_/KI-etch bath. As Figure 2c shows, the optical microscope image depicts the underlying gold substrate within the irradiation areas unaffected by the etching process as evidence that the crosslinked mechanism take place in the BPD-Ni SAM after radiation, although it was etched within the non-irradiated region.

#### Fabrication of the top electrode

**Figure 3:**
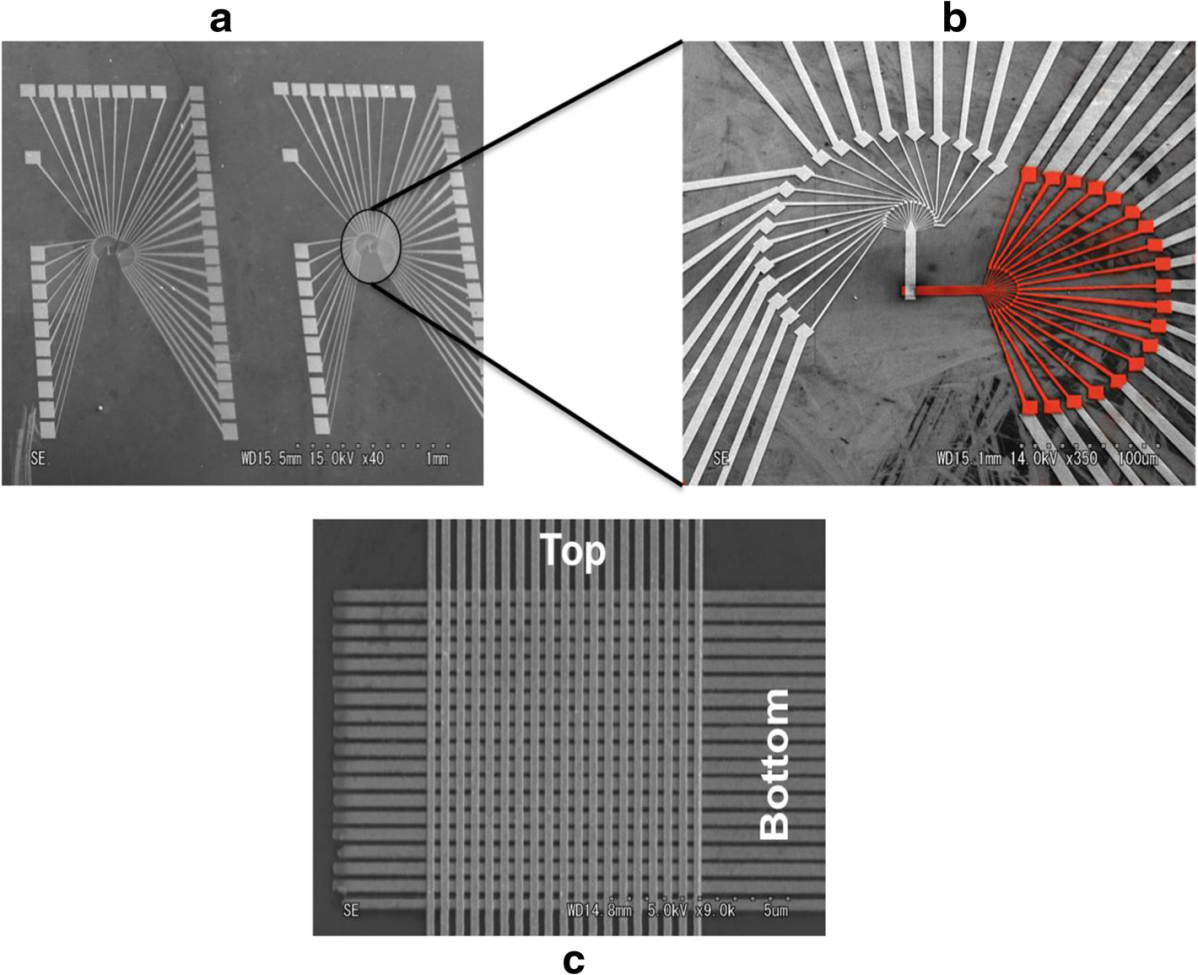
**SEM images of the crossbar device. (a)** General view of the two devices. **(b)** Red structure shows the bottom electrodes. **(c)** High-magnification images of the crossbar device to show the bottom and the top electrodes.

Pre-patterning resist for the top contact was accomplished similar to the fabrication of the bottom electrode. First, PMMA 950 was spin-coated at 2,000 rpm for 90 s and baked at 180°C for 3 min. Then ESPACER 300Z™ (Showa Denko K.K.) was spin-coated on top of the PMMA at 2,000 rpm for 60 s. The 100-nm bar patterns perpendicularly aligned with respect to the bottom electrodes were fabricated using the electron beam lithography (50 kV, 100 mC/cm^2^). Then the resist was developed in the MIBK-IPA solution for 30 to 40 s to form the pattern for the top electrode lines. Finally, 10 nm of titanium and 150 nm of gold were deposited by electron-beam evaporation on the photoresist-patterned wafer. The wafer was immersed in acetone to remove the photoresist and the excess metal which adhered on the resist (Figure 1e). Figure 3 depicts SEM images of the crossbar devices.

### Characterization of crossbar devices

Temperature-dependent *I*-*V* characteristics of the molecular devices were acquired using a standard semiconductor parameter analyzer (HP 4145 B; Agilent Technologies, Sta. Clara, CA, USA) on a cryostat modulated between 8 K and room temperature to probe the respective pads for the top and bottom contacts to the molecule.

### DFT calculations

Density functional theory (DFT) calculations were conducted using ORCA [13]. The PBE0 [14] was used in combination with triple-zeta plus polarization basis set (Ahlrichs TZV (2*df*, 2*pd*)) [15].

## Results and discussion

### SAM properties

**Figure 4:**
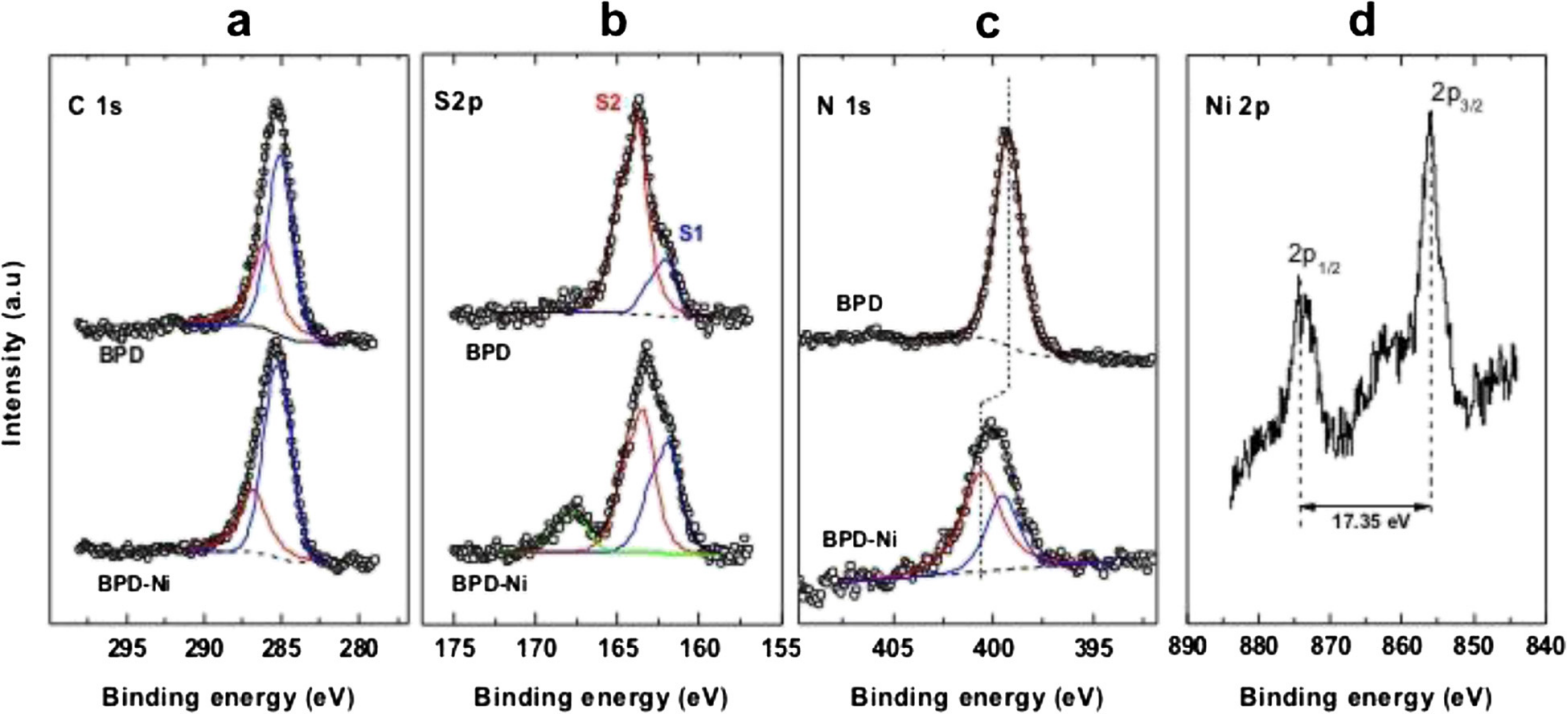
**XPS of: a) C 1 *s***, **b) S 2*p***, **c) N 1 *s*, and d) Ni 2 *p *spectra of the BPD and BPD-Ni crosslinked SAMs on gold.** Some spectra are decomposed into the individual contribution related to different species; see text for details.

The BPD SAM on gold was characterized using XPS. The C 1 *s*, N 1 *s*, S 2*p*, and Ni 2*p* XPS spectra are portrayed in Figure 3. The C 1 *s* spectrum shows that the main peak at 285.5 eV is a superposition of the contribution from different carbons: the aliphatic (CH_2_) and the C = C moieties at the low binding energy (the blue line in Figure 4a). And the C in the rings directly bound to the nitrogen atoms of the pyridine unit at the high binding energy (red line in Figure 4a) [16].

The spectral deconvolution of the S 2*p* BPD SAM (Figure 4b) was performed as usual, setting a 1.2 eV 2*p*_1/2,3/2_ splitting and here introducing two doublets: the first at 162 eV S_1_ (S 2*p*_1/2_) is commonly assigned to the thiolate species, which indicates that the molecules in the BPD films are attached to the substrate via the thiolate. The second doublet is at about 163.5 eV S_2_ (S 2*p*_3/2_) corresponding to sulfur of the free thiol (SH) groups or S-S bonds [4,5]. The N 1 *s* XPS spectra of the BPD SAM are displayed in Figure 4c. A single symmetric peak at 399 eV is assigned to the nitrogen in the pyridine rings. Thickness of the BPD film calculated from the carbon to Au XPS signal ratio using the dodecanethiol (DDT) SAM as reference is approximately 2.4 nm, which shows good agreement with the BPD molecule height.

Treatment of the BPD SAM with NiCl_2_ brings a significant change in the S 2*p* and the N 1 *s* spectra. The S 2*p* spectra (Figure 4b) show a clear change in the relative intensity of both components S_1_ and S_2_ after exposure to Ni. The S_1_ component increases significantly. On the other hand, the intensity of the free S (S_2_ peak) at the SAM interface decreases in intensity after exposure to Ni, which is probably attributable to the formation of the Ni thiolate species at the SAM-ambient interface [17,18]. In this experiment, the total eradication of the S_2_ was not achieved, which indicates a partial formation of the Ni thiolate species at the SAM-ambient interface. In addition, it is noteworthy that the dithiol SAMs are extremely sensitive to photo-oxidation [4,6]. Solutions that are well-degassed by Ar and the absence of ambient light during the preparation steps can minimize oxidation. The peak at 168 eV was assigned to the partial formation of the sulfonate at the interface, which was probably produced during the cleaning and transfer of the samples.

Regarding the N 1 *s* spectra (Figure 4), the addition of Ni produces a chemical shift of the main peak to a higher binding energy by 1.2 eV, which is a fingerprint of the binding of the N of bipyridine to the Ni^2+^ moiety [19,20].

Figure 4d shows the Ni 2*p*_3/2_ region. The peak at 855.9 eV is assigned to Ni^2+^. The shake-up structure and the energy separation of 17.49 eV between the 2*p*_3/2_ and 2*p*_1/2_ peaks are consistent with divalent Ni [21,22].

### *I*-*V* characteristics

**Figure 5:**
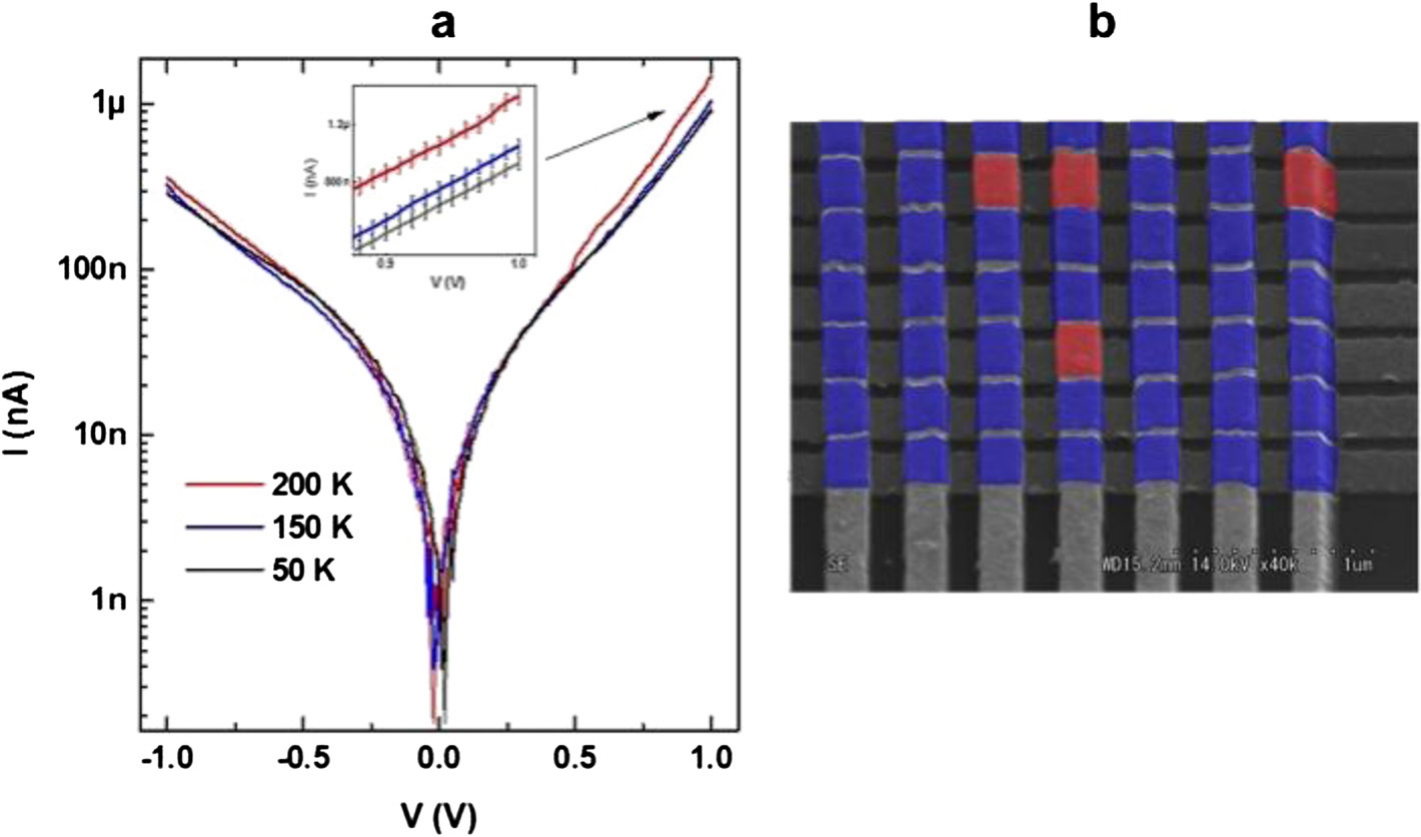
***I*- *V *characteristics of crosslinked molecular devices. (a)** Set of temperature-dependent *I*-*V* between the top and bottom electrodes. The vertical bars indicate the data dispersion related to sample-to-sample variations **(b)** Data for 49 junctions: blue areas show non-shorting junctions. Red areas show defective junctions.

The electrical behavior of the crosslinked molecular devices was studied by testing each crosswire molecular device junction (Figure 5a). The electrical measurements of the gold-BPD-Ni^2+^-Ti-Au junctions show good stability and reproducible current values. As described above, when the second electrode is evaporated on the top of the self-assembled monolayer, it is well known that the metal atoms might penetrate the molecular film and short-circuit the device. The high fidelity of the crossbar devices (see Figure 5b) represented in this work is probably the result of appropriate engineering of the film and the electrodes: (i) the higher packing density of the SAM and the crosslinking strategy enhance the resistance to metal atom diffusion processes that occur during the evaporation of the top electrodes; and (ii) by decreasing the area covered by the bottom electrodes (100 nm), the probability of defects is reduced.

The temperature-dependent *I*-*V* characteristics of devices composed of gold-BPD-Ni^2+^-Ti-gold were studied at temperatures of 50 to 200 K. This study was undertaken to distinguish between transport attributable to molecular phenomena and transport involving metal filaments [23]. The electron transport mechanism of the crosslinked monolayer of the BPD-Ni^2+^ in this nanocrossbar device at temperatures of 50 to 200 K shows a decrease in the current with decreasing the temperature, as might be expected for thermally activated hopping transport [24].

The temperature-dependent *I*-*V* characteristics of the crosslinked BPD-Ni^2+^ SAM at the crossbar junctions show two transport regimes. The first regime is direct tunneling (coherent), which happens at low bias where the *I*-*V* is rather insensitive to temperature. They only differ in terms of voltage dependence [25]. The second regime, regarded as hopping conduction, happens above 0.48 V. It is a thermally activated process that is sensitive to temperature.

**Figure 6:**
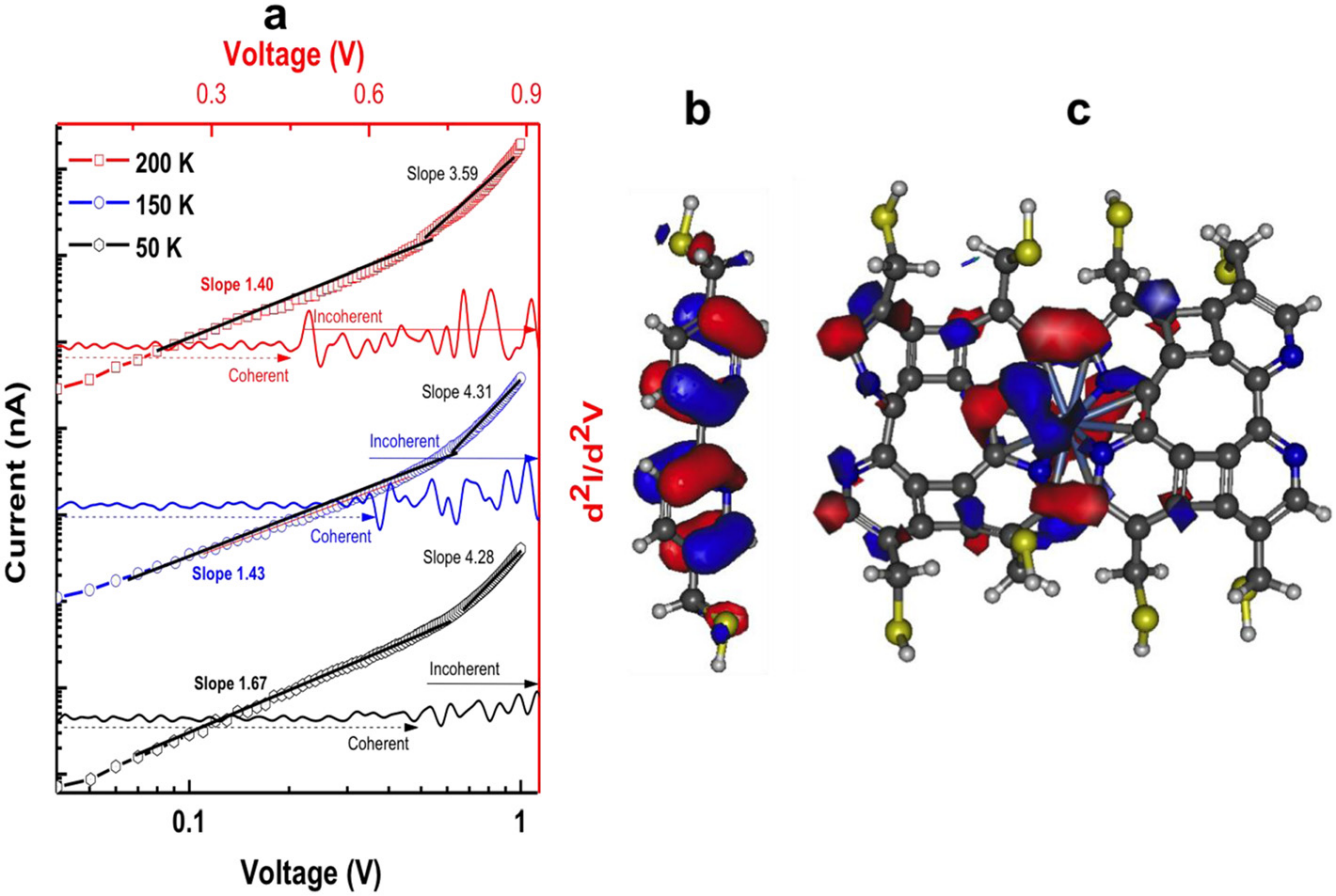
**Log( *I *)-log( *V *) plot of the *I *- *V *characteristics and electronic structure of BPD and crosslinked BPD-Ni SAM. (a)** Temperature-dependent *d*^2^*i*/*d*^2^*v* versus voltage and the log(*I*)-log(*V*) plot of the *I*-*V* characteristics. **(b, c)** Electronic structure of the BPD and the crosslinked BPD-Ni SAM as computed from DFT (see the text).

The study of log(*I*)*-*log(*V*) plot of the *I-V* characteristics and the *d*^2^*i*/*d*^2^*v* versus voltage provides key information related to the transport mode of the molecules on metallic junctions [24]. Figure 6a shows recorded traces of the temperature-dependent *d*^2^*i*/*d*^2^*v* versus voltage and the log(*I*)*-*log(*V*) plot of the *I-V* characteristics of the crosslinked BPD-Ni^2+^ on the crossbar devices. It is possible to discern different transport regimes as a function of the voltage using the log(*I*)*-*log(*V*) plot of the *I-V* characteristics. At the low-voltage bias, the plots are linear with a slope of about 1.45 for the different temperature. The crossbar architectures exhibit a second regime at the voltage higher then 0.45 with slope of about 4.31.

The *d*^*2*^*i/d*^*2*^*v* shows different peaks located at the near-infrared region [26,27]. A possible explanation for this observation can be sought in the electronic properties of the crosslinked SAM. Figure 6b,c presents frontier orbitals of the BPD and the crosslinked BPD-Ni structures as obtained from a DFT calculation of the isolated molecules. The highest occupied molecular orbital (HOMO) electronic density distribution shows localization of the electrons on the bipyridine in both cases. It is possible that when an electron proceeds through the valence orbitals (HOMO), it can also be coupled to the single local vibrational mode of the pyridine at the corresponding voltage bias. It is noteworthy that different molecular electronic studies show that involvement of the valence bond in such phenomena remains unclear [24,28].

The temperature-dependent *d*^2^*i*/*d*^2^*v* versus voltage characteristics shows a clear impact of temperature on transport properties. High temperatures favor incoherent transport. However, low temperatures favor the coherent mode (Figure 6a). These phenomena are explainable by the impact of electron vibration (phonon) interaction [24,28]. The high temperature reduces the inelastic scattering length by increasing the phonon population, rendering electron-phonon interaction sufficiently strong to activate the different vibrational mode of the molecular system, which can engender pronounced current. This regime, called incoherent, is usually designated as hopping. This phenomenon was explained in an earlier report [28], which presented data similar to those from the present study, with junctions fabricated using the electromigration technique.

## Conclusions

This report presents a novel method to produce a molecular electronic crossbar device basing in two strategies to avoid penetration of the metal through the organic film: (i) using the crosslinked self-assembled monolayer of 5,5′-bis (mercaptomethyl)-2,2′-bipyridine-Ni^2+^ (BPD-Ni^2+^) on a gold surface and (ii) by reducing the area of the bottom electrodes (100 nm), the probability of the SAM defects is reduced.

Temperature-dependent *I-V* characteristics of devices show thermally activated hopping transport excluding existence of spurious metal filament transport. Further studies are in progress in our institute to elucidate the electron transfer mechanism in junctions based on a crosslinked SAM.

## Competing interests

The author declares that he has no competing interest.
